# Bleeding and thrombosis in a patient with primary antiphospholipid syndrome using norethisterone: a case report

**DOI:** 10.1186/s13256-015-0554-3

**Published:** 2015-04-22

**Authors:** Sawsan Abdullah Al Abdulhai, Mahmoud Wahid El-Ali, Mohsen El-Sherbiny El-Dahshan

**Affiliations:** 1 Department of Rheumatology, Dammam Medical Complex, Dammam, Saudi Arabia; 2 Department of Hematology, Dammam Medical Complex, Dammam, Saudi Arabia; 3 Department of Radiology, Dammam Medical Complex, Dammam, Saudi Arabia

**Keywords:** Antiphospholipid syndrome, Norethisterone, Bleeding, Thrombosis

## Abstract

**Introduction:**

Antiphospholipid syndrome is known to be associated with the occurrence of venous and/or arterial thrombosis. There are several factors that might trigger the risk of thrombosis in antiphospholipid syndrome, including drugs, however bleeding is rare. Only a few cases of antiphospholipid syndrome have reported simultaneous bleeding and thrombosis, and only a few of these cases have reported thrombosis induced by norethisterone when used by patients with an underlying risk factor for thromboembolism.

**Case presentation:**

We report the case of a 35-year-old Saudi woman diagnosed with antiphospholipid syndrome with a history of several spontaneous miscarriages and two previous lower limb deep vein thromboses. She had used norethisterone to postpone her menstruation and presented to our institution with severe menorrhagia. During admission, she developed thrombocytopenia, and at the same time she was found to have extensive inferior vena cava and bilateral common iliac thrombosis.

**Conclusions:**

This case report is of interest to rheumatologists, hematologists and radiologists because we have found that the presence of bleeding and thrombocytopenia do not preclude the concomitant occurrence of thrombotic complications of antiphospholipid syndrome. Norethisterone is normally safe to take, but it is not suitable for patients with an increased risk of deep vein thrombosis. Also, the simultaneous management of thrombosis and heavy vaginal bleeding is a challenge for clinicians since there are no evidence-based guidelines regarding the management of these patients.

## Introduction

Antiphospholipid syndrome (APS) is defined by two major components: the occurrence of at least one clinical feature, venous or arterial thrombosis and/or pregnancy morbidity, and the presence of at least one type of autoantibody known as an antiphospholipid antibody ((aPL), detected by lupus anticoagulant tests, anticardiolipin and/or anti-β2 glycoprotein-I antibodies on two separate occasions, at least 12 weeks apart. Other aPLs, such as antibodies to prothrombin, annexin V, phosphatidylserine and other proteins, have also been associated with APS. However, the understanding of the potential roles played by such antibodies in APS is incomplete, and assays for these antibodies are not a part of the standard evaluation when APS is suspected. Antibodies to prothrombin are associated with bleeding and thrombosis [1]. Thrombosis is the most common clinical manifestation of APS, and the recurrence of thrombotic events is common as well. Thrombosis in APS can happen either spontaneously or with a triggering factor. Another common clinical manifestation of APS is thrombocytopenia [2], but this does not preclude the occurrence of thrombotic complications of APS. It is common to treat thrombosis associated with APS, but it is quite difficult to treat thrombosis and bleeding at the same time. In the literature, only a few cases of APS have reported simultaneous bleeding and thrombosis [3-8], and only a few of these cases have reported thrombosis induced by norethisterone when used by patients with an underlying risk factor for thromboembolism [9].

Norethisterone contains a hormone similar to the progestogen hormone produced naturally in the body. Despite the number of menstrual cycle disorders on its licensed indications [10], norethisterone is prescribed by most clinicians to stop heavy menstrual bleeding events [11]. Before 1999, it was thought that high-dose norethisterone was free of serious adverse effects, but two studies have revealed an increased incidence of venous thromboembolism (VTE) in women taking high-dose oral progestogens, either because progestogen increased VTE risk or because these women had an underlying risk of VTE [11]. These risk factors have been added to the contraindications of norethisterone [12,13].

## Case presentation

We report the case of a 35-year-old Saudi woman, living in the Eastern Province; a three-hour drive from our hospital. She was a house wife, married for 12 years, with no children. When she was admitted to our hospital, she was complaining of abdominal pain for 10 days and menorrhagia for five days. She was first seen at our hospital’s rheumatology department three months prior to her admission, and was diagnosed at her local hospital with APS five years prior to her presentation. During her follow-up visits at her local hospital she had two deep vein thromboses (DVTs). The first was of her proximal right lower limb, for which she received heparin followed by warfarin, and the second was of her distal right lower limb. As she was two-and-a-half months pregnant at the time of her second thrombosis, she was given enoxaparin subcutaneously. She had a history of nine miscarriages over 12 years, all at a gestational age of one-and-a-half to two-and-a-half months. In her first to fifth pregnancies, she was not receiving any anticoagulation treatment, but she did receive anticoagulation treatment during subsequent pregnancies, and 81mg of aspirin per day. She also had a history of arthralgia, fatigue and thrombocytopenia four years previous, with a platelet count of 45 to 50×10^3^. However, there was no history of mouth ulcer, skin rash, photosensitivity, renal disease or convulsion, or any other medical illness.

At 13 days before her admission, she was planning to travel, prior to which she started using norethisterone (oral progestogen) 5mg two to three times per day to delay her menses during the trip. The medication was not prescribed to her by a physician, nor did she discuss it with any physician before she started using it; rather she bought the drug herself. Three days after her arrival to her destination, which is two hours away by airplane, she developed abdominal pain mainly in the epigastric area, in the form of cramps, associated with nausea and vomiting, but she had no hematemesis and no presentation of diarrhea or fever. During that time, she stopped taking norethisterone, which she had been using for a total of 15 days, and started having severe vaginal bleeding with clots. Because of these symptoms she interrupted her vacation and returned to Saudi Arabia, where she was admitted to her local hospital for three days. She left her local hospital against medical advice, to be admitted to our hospital. Her social history revealed that because of her inability to have children, her husband was considering having another wife, which was a cause of stress.

During her examination she was conscious, oriented, pale and not jaundice. Her temperature was 37°C, her pulse rate was 120 per minute, her blood pressure was 120/80 and her respiratory rate was 22 breaths per minute. Her abdomen was soft, but only tender at the epigastric and the right upper quadrant. Her lower limbs showed no signs of DVT and her other systemic examinations were normal.

Table 1 shows the baseline lab tests at her first presentation. Her iron levels were at 140ug/dL (normal level: 37 to 145), which suggested possible α thalassemia. Even though she was currently receiving warfarin, her PTT levels were still high (PTT = 123.4 seconds (control 34)), suggesting the presence of lupus anticoagulant. She showed a mild positive resistance to activated protein C (APC-resistance ratio of 0.76, normal levels are between 0.8 and 1.1), which may have been due to anticoagulation. Lupus anticoagulant, protein C and protein S tests were not done because she was already on warfarin.

**Table 1:** **Laboratory data at first presentation**

**Lab tests**	**Reference range**	**During first clinic visit**	**Repeated**
WBC (×10^3^/μL)	4-11×10^3^	5.63×10^3^	
Hemoglobin (g/dL)	12-14	11.8	
MCV (fl)	86-96	64	
MCH (pg)	27-32	20	
Platelet (×10^3^/μL)	150-450	315×10^3^	
Ferritin (ng/mL)	13-150	24	
S.Iron (ug/dL)	37-145	140	
PT (seconds)	Control 13	27	
PTT (seconds)	Control 34	123.4	
INR (seconds)	0.8-1.2	2.7	
Urea (mg/dL)	15-38	29	
Creatinine (mg/dL)	0.6-1	1.04	
C3 (mg/dL)	80-152	142	
C4 (mg/dL)	14-40	28.6	
ANA titer		1:320	
Anti-DNA		Negative	
Anti-Sm EU/mL	Negative <20	24.4	
	Positive >25		
Anti-SSA(Ro) EU/mL		Negative	
Anti-SSB(La) EU/mL		Negative	
Anti-cardiolipin:			
(screening)	<20U/mL	281.75	388.9
IgG		N/A	176.2
IgM		N/A	
Anti-β2 GPI antibodies:			
IgG	<20U/mL	182.9	343.7
IgM	<20U/mL	N/A	N/A
Lupus anticoagulant	Not done (Patient on warfarin)		
Protein C	Not done (Patient on warfarin)		
Protein S	Not done (Patient on warfarin)		
APC-resistance ratio	0.8-1.1	0.76	
Anti-thrombin Ш	75-120	88.6%	

White blood cells (WBC), mean corpuscular volume (MCV), mean corpuscular hemoglobin (MCH), serum iron (s. iron), Prothrombin Time (PT), Partial Thromboplastin Time (PTT), International Normalized Ratio (INR), antinuclear antibody (ANA), antibodies to double-stranded (Anti-DNA), Antibody to the smith antigen (Anti-Sm), anti-Ro antibodies (Anti-SSA), anti-La antibodies (Anti-SSB), Immunoglobulin-G (IgG), Immunoglobulin M (IgM), activated protein C resistance ratio (APC-resistance ratio), not available in the laboratory (NA).

Table 2 shows the tests conducted on her first day of admission. She had an acute drop from her baseline hemoglobin level to 6.6g/dL. Coagulation studies demonstrated a markedly prolonged PT of 173 seconds and INR of 8.36 seconds, and PTT with no clotting. Her peripheral blood film showed microcytic, hypochromic red blood cells with normal white blood cells count, however her platelets were decreased and marked platelet anisocytosis was seen. Her repeat peripheral blood film conducted on 7 June 2011 showed marked anisocytosis with microcytes, occasionally fragmented forms, neutrophilic leukocytosis and marked thrombocytopenia. Table 3 shows the repeated complete blood count, coagulation profile and renal function tests during the patient’s subsequent admission days and three months after her discharge. The tests showed leukocytosis which responded to antibiotics. The tests also showed thrombocytopenia, which improved with intravenous immunoglobulin and methylprednisolone.

**Table 2:** **Laboratory data obtained upon admission to the hospital**

**Lab tests**	**Reference range**	**Day 1**
WBC (×10^3^/μL)	4-11×10^3^	7.02
Hemoglobin (g/dL)	12-14	6.6
Platelet (×10^3^/μL)	150-450	85×10^3^
PT (seconds)	13.3	173
INR (seconds)	0.8-1.2	8.36 (high)
PTT (seconds)	33.7	No clot
LFT		Normal
S. albumin (g/dL)	3.5-5	2.2
S. Protein (g/dL)	6.4-8.2	6.7
S. amylase (IU/L)	10-220	32
S. lipase		N/A
Urea (mg/dL)	15-38	55
Creatinine (mg/dL)	0.6-1	1.5
Urine analysis:		
Albumin		Nil
Pus cells		0
RBC		1-2
C3	80-152	139
C4	14-40	22.7
Anti-DNA		Negative
Pregnancy test		Negative
24 hour urine:		
Protein (mg/dL)	<149	228
Creatinine clearance	88-128mL/min	117

White blood cells (WBC), Prothrombin time (PT), International Normalized Ratio (INR), Partial Thromboplastin Time (PTT), Liver function test (LFT), serum albumin (S. albumin), serum protein (S. protein), serum amylase (S. amylase), serum lipase (S. lipase), red blood cells in urine (RBC), autoantibodies (Auto Abs), antibodies to double-stranded (Anti-DNA).

**Table 3:** **Summary of lab tests during admission and three months after discharge**

**Lab test**	**Reference range**	**Day 1**	**Day 5**	**Day 6**	**Day 7**	**Day 8**	**Day 10**	**Day 12**	**OPD (3 months later)**
**WBC**	4-10x10^3^	7.02	6.97	**14.15**		7.7	**11.75**	10.75	10
**Hb**	12-16g/dl	**6.6**	10.5	9.9		**8.8**	**8.7**	**9.5**	12.8
**MCV**	86-96FL	**60**	**62**	**61.8**					
**MCH**	27-32Pg	20	20	20					
**PLATELET×10** ^**3**^ **/μL**	150-400	**85X10** ^**3**^	**42X10** ^**3**^	**31X10** ^**3**^		**39X10** ^**3**^		**96x10** ^**3**^	419
**PT**	13 seconds	**173**	15.4	17.5	27.3	48.9	37.9		
**INR**	0.8-1.2 seconds	**8.36**	1.4	1.6	2.6	4.8	3.5		
**PTT**	34 seconds	**no clot**	81.6	65.9	83.3	83.5	88.3		
**AST**	0-37IU/L	45	**208**	**75**				24	11
**ALT**	0-40IU/L	**67**	**124**	**99**				27	22
**ALP**	39-117IU/L	109	138	134				67	77
**Total Protein**	6.6-8.7g/dl	6.7	7.5	7.2				6.57	7.6
**S. Albumin**	3.5-5.0g/dl	**2.2**	2.3	2.1				2.2	4.46
**T.BILI**	0-1Mg/dl	0.8	**1.9**	0.8				0.88	
**D.BILI**	0-0.2g/dl	0.46	**1.31**	0.5				0.26	
**LDH**	105-333IU/L			346					
**Urea**	15-38Mg/dl	48		30				45	38
**Creatinine**	0.6-1Mg/dl	**1.2**		0.9				0.93	0.8

Values in bold indicate abnormal levels.

White Blood Cells (WBC), Hemoglobin (Hb), Mean Corpuscular Volume (MCV), Mean Corpuscular Hemoglobin (MCH), Prothrombin Time (PT), International Normalized Ratio (INR), Partial Thromboplastin Time (PTT), Aspartate Aminotransferase Test (AST), Alanine Aminotransferase Test (ALT), Alkaline Phosphatase (ALP), Total Protein (TP), serum albumin (S. Albumin), Total Bilirubin (T.BILI), Direct Bilirubin (D.BILI), Lactate Dehydrogenase (LDH).

The initial diagnosis was APS, gastritis or food poisoning, with menorrhagia and anemia secondary to withdrawal of norethisterone in a patient using warfarin.

During admission, she received a total of four units of packed red blood cells and four units of fresh frozen plasma. She also became febrile, with a temperature of 38.8°C. On the second day of admission, she was started on 60mg of enoxaparin administered subcutaneously every 12 hours, 3mg of warfarin once per day (on the third day of admission, which was later adjusted according to INR), a course of an antibiotic (ceftriaxone), 20mg of omeprazole per day and butylscoplamine bromide for upper abdominal pain. Her previous medications were continued, which consisted of hydroxychloroquine 200mg twice per day, calcium carbonate and vitamin D. She had had no vomiting since her admission, but continued to complain of upper abdominal pain in the following days, in spite of receiving different analgesics. Her abdomen continued to be soft, and only tender at the epigastric and the right upper quadrant. Her upper gastrointestinal endoscopy revealed moderate erosive gastritis, which could not explain her pain. Her pelvic and abdominal ultrasound only showed mild hepatomegaly, but was otherwise normal. Her Doppler abdominal ultrasound showed no venous thrombosis.

**Figure 1:**
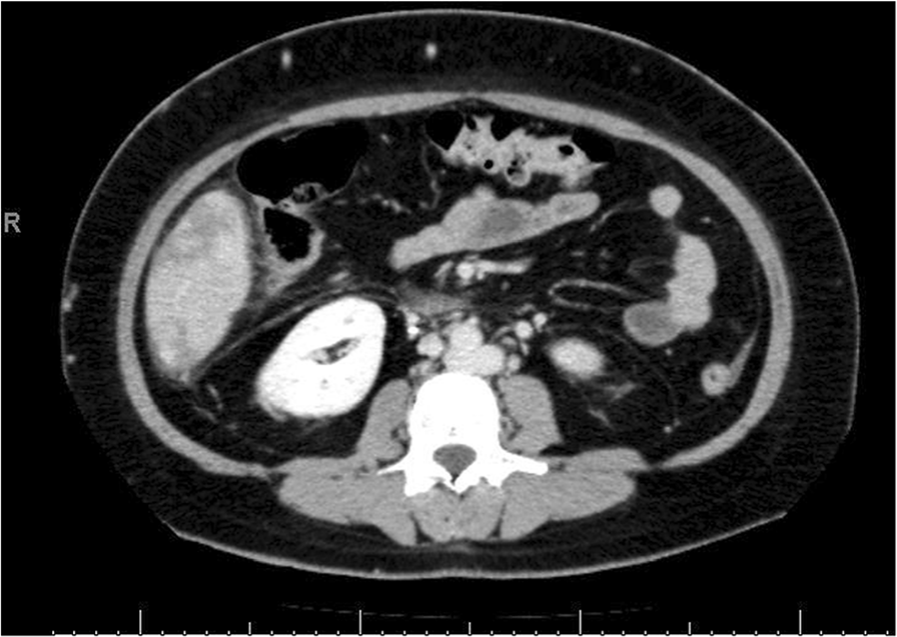
Computed tomography scan showing an abnormal perfusion with multiple small ill-defined hypodensities of the right hepatic lobe.

**Figure 2:**
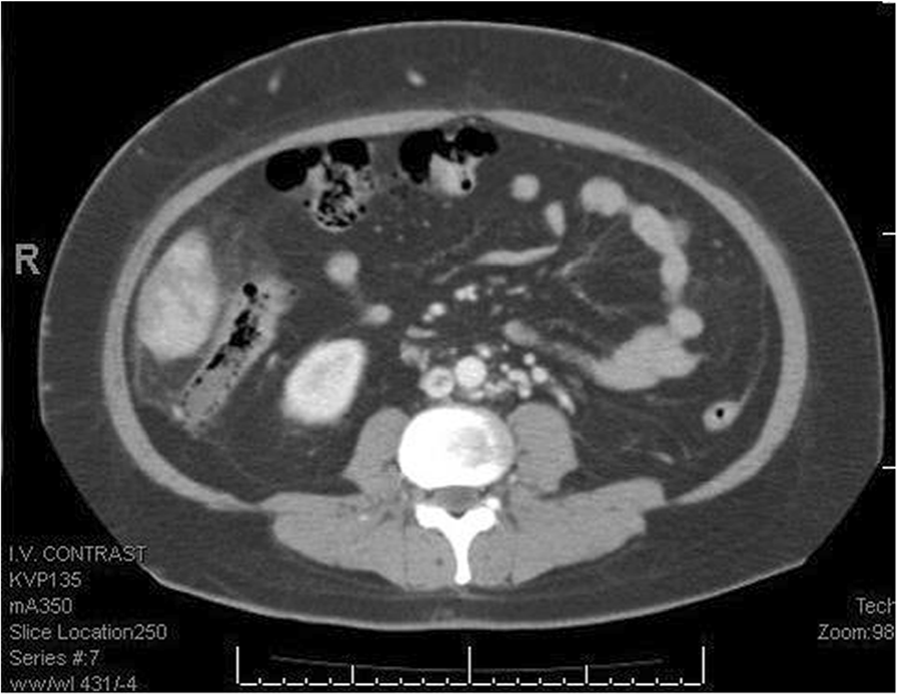
Computed tomography scan of the abdomen showing a clot within the inferior vena cava.

**Figure 3:**
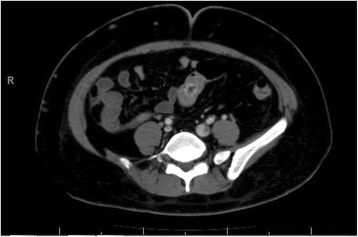
Computed tomography scan revealing the extension of the clot to the common iliac veins.

**Figure 4:**
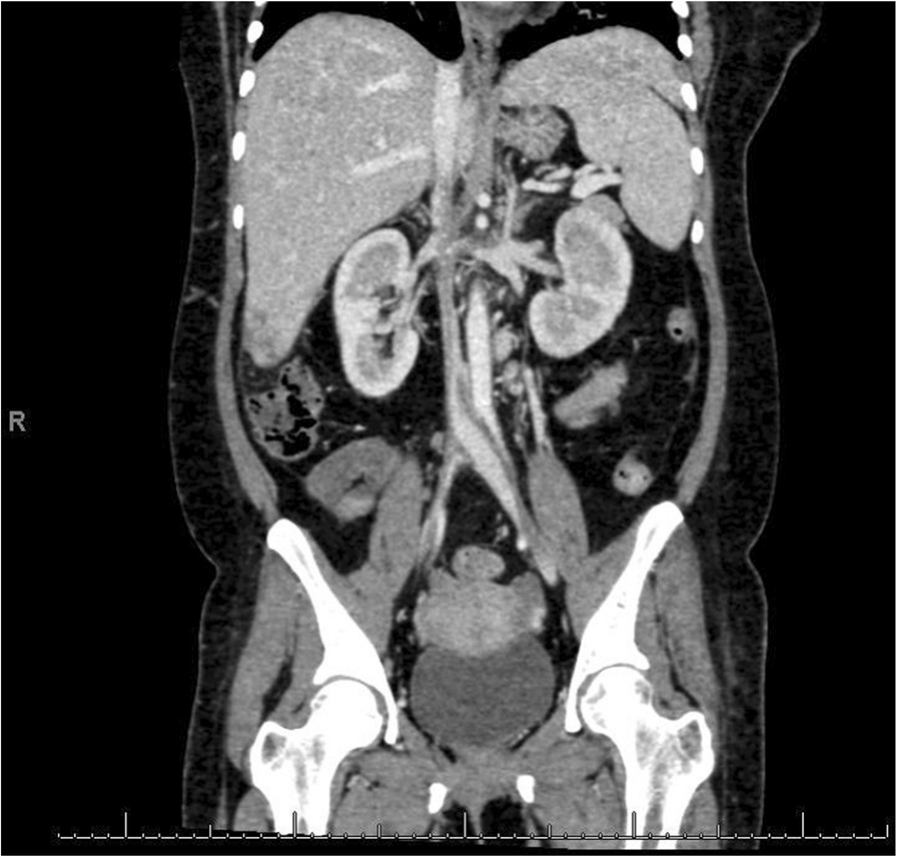
Computed tomography scan showing the extent of the inferior vena cava thrombus to the iliac and the distal left renal vein.

At that stage, it was suspected that there was a psychological contribution to her expression of pain, which was suspected due to her social history. However, investigations were continued by conducting a triphasic abdominal computed tomography (CT) scan, which showed hepatomegaly with multiple small ill-defined hypodensities predominantly in her right lower liver and sub-diaphragmatic region. These are shown in the contrast-enhanced CT scans in Figures 1, 2, 3 and 4. The radiologist identified those lesions as an infarct secondary to vasculitis. The inferior vena cava (IVC) was narrowed in the subhepatic region and showed a filling defect in the distal segment, which extended into the right and left common iliac and external iliac veins, with prominent azygos. Her bilateral lower limb venous Doppler ultrasound showed no abnormality.

On her sixth day of admission a dose of 1g per day of intravenous methylprednisolone was given for three days, followed by 60mg per day of oral prednisolone. She also received 0.4g/kg per day of intravenous immunoglobulin (IVIG) for four days. Later on, the enoxaparin was stopped, and the warfarin dose was adjusted according to the INR. Her vaginal bleeding was initially heavy with clots, but it gradually decreased until it stopped completely. After she was discharged, the oral prednisolone was tapered gradually. Her previous medications were continued, which included warfarin,81mg aspirin,hydroxychloroquine 200mg twice per day, calcium carbonate and vitamin D.

**Figure 5:**
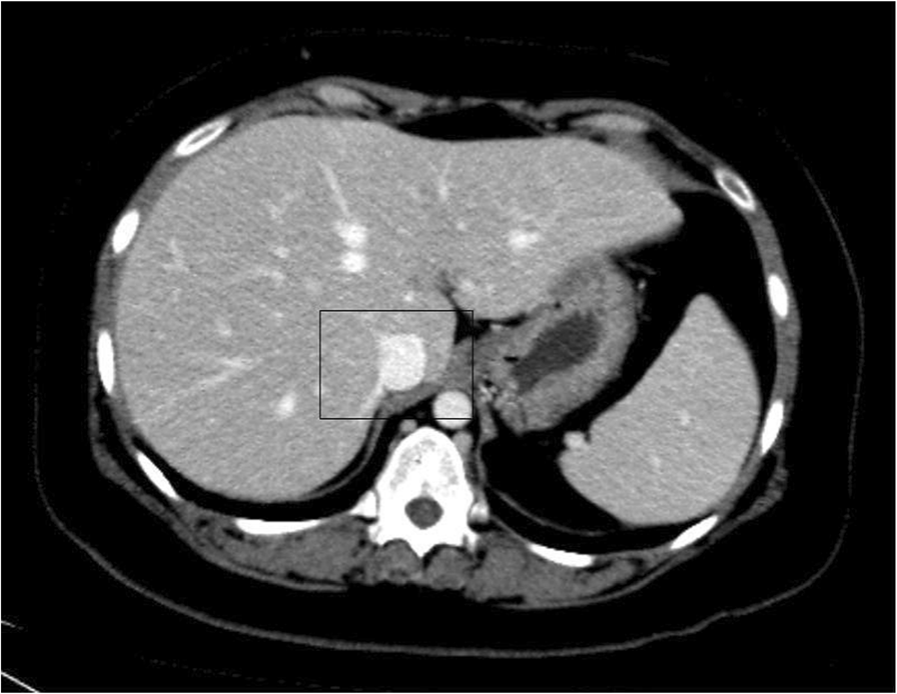
Computed tomography scan showing a normal intra-hepatic portion of the inferior vena cava (boxed region).

**Figure 6:**
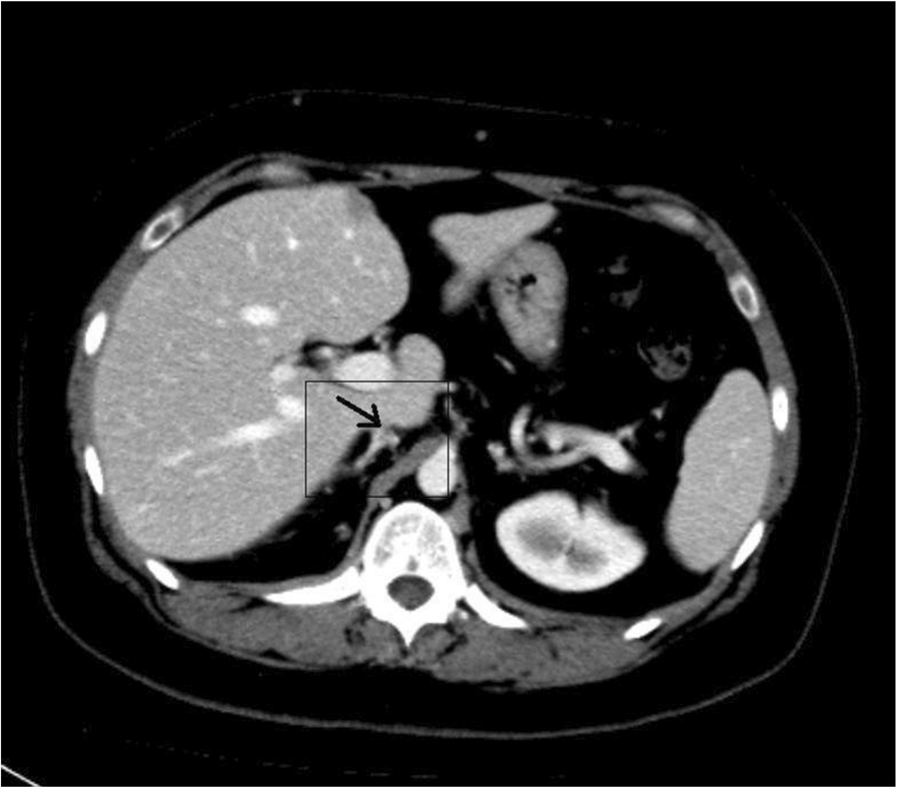
Computed tomography showed narrowed infra hepatic IVC (boxed region and arrow).

**Figure 7:**
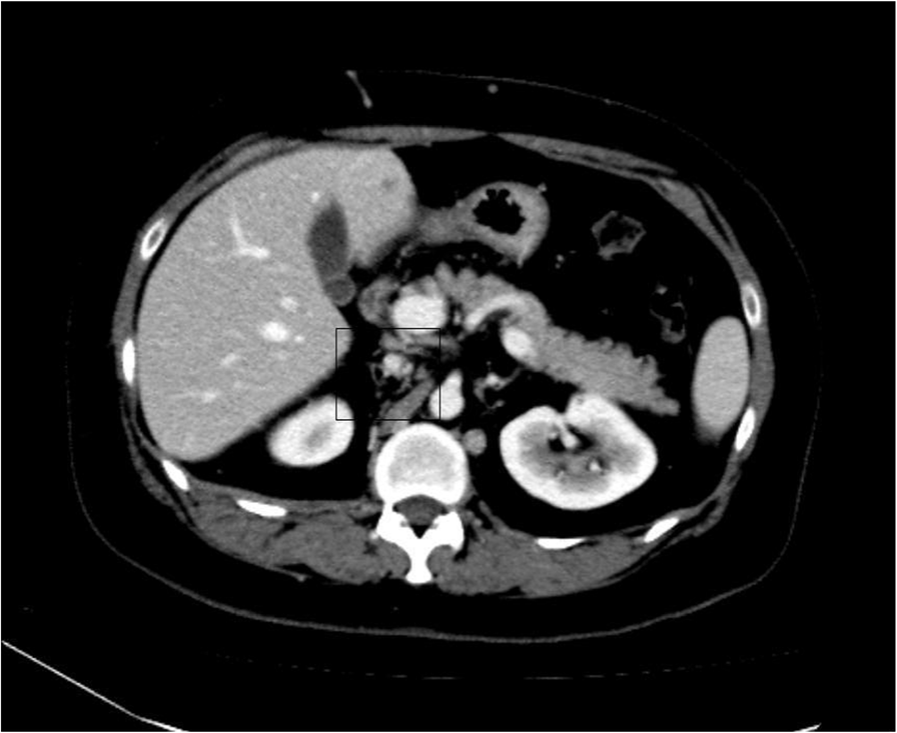
Computed tomography scan showing the narrowed inferior vena cava distal to the liver and dilated collaterals on the anterior abdominal wall (boxed region).

**Figure 8:**
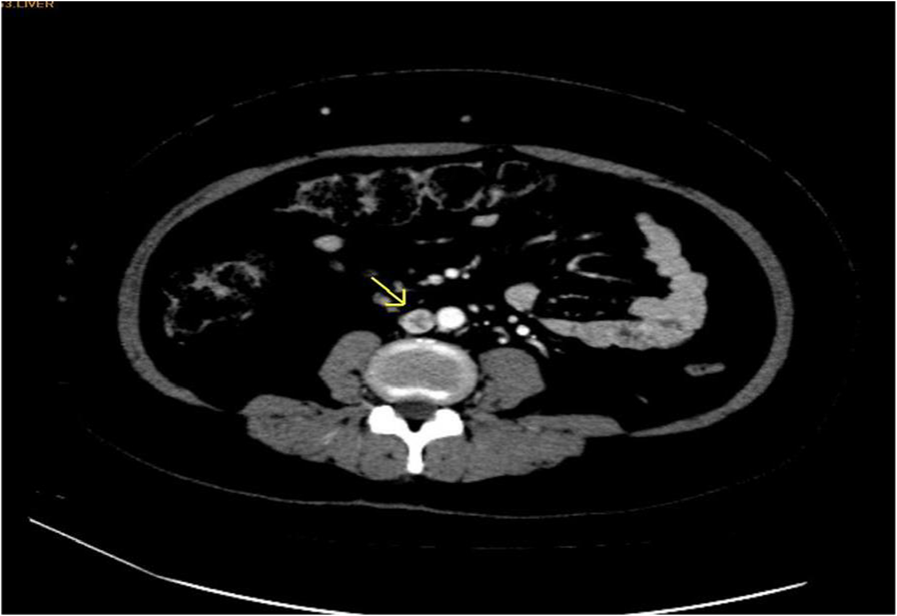
Computed tomography scan at the level of the renal hilum showing narrowing and thrombosis of the inferior vena cava (arrow).

**Figure 9:**
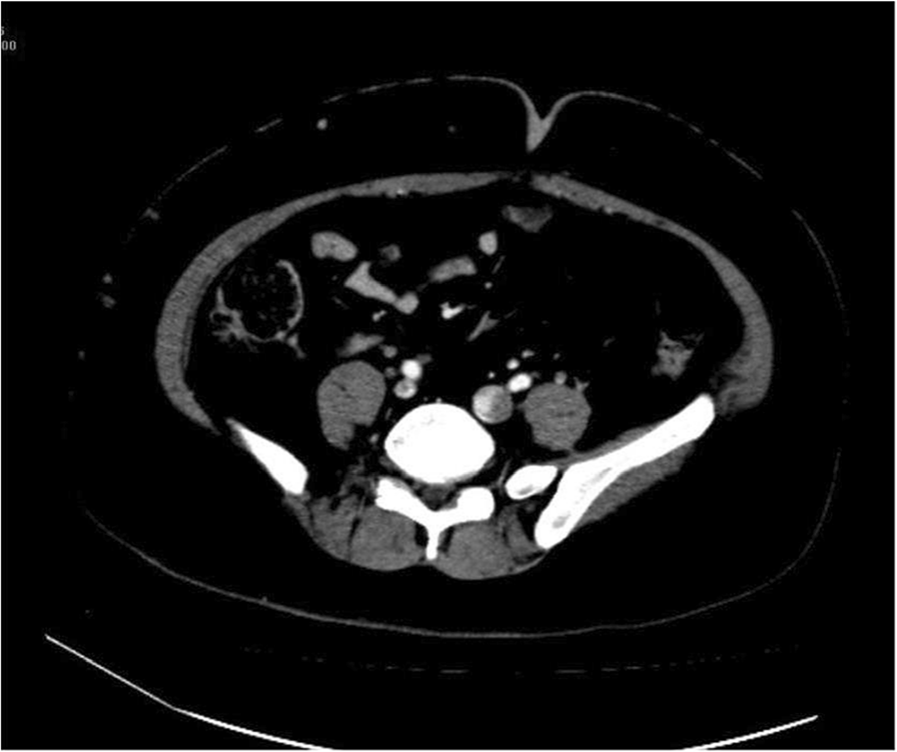
Computed tomography scan below the level of the kidneys showing a filling defect in the inferior vena cava.

**Figure 10:**
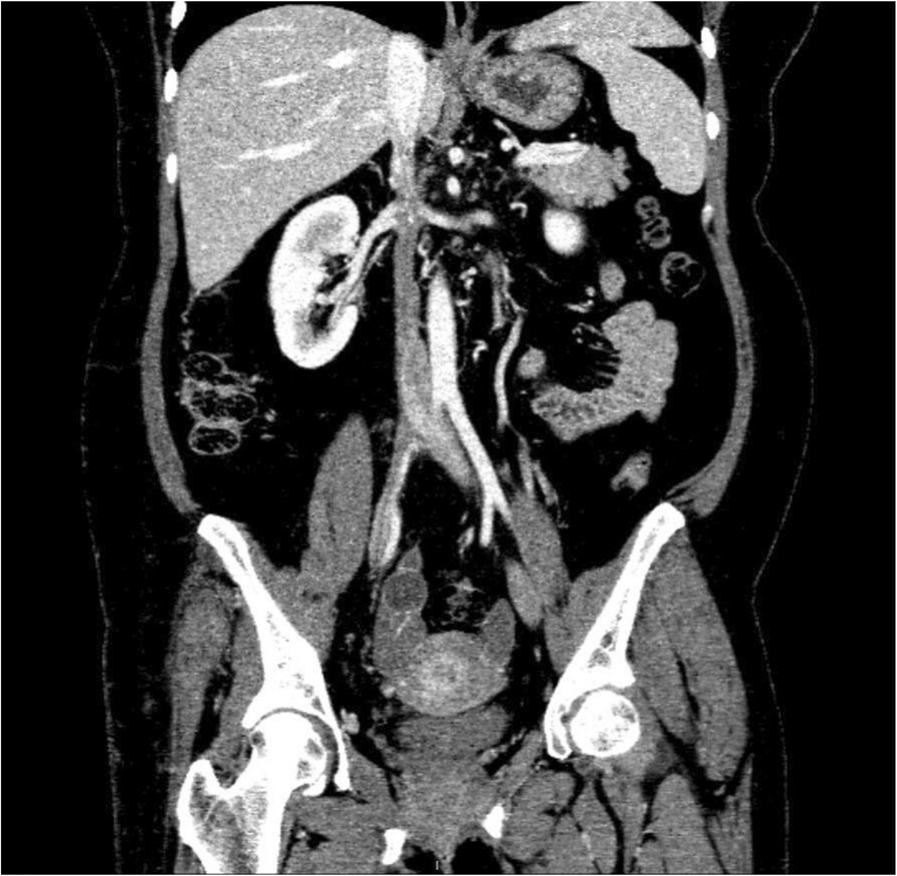
Computed tomography scan showing extensive thrombosis of the inferior vena cava.

The final diagnosis was APS, menorrhagia, anemia and venous thrombosis, possibly triggered by norethisterone. Her outpatient department (OPD) follow-up visit showed that she was doing well. Her follow-up abdominal triphasic CT scan was conducted three months later, which showed only two hypodense lesions in the liver, which were most likely fatty infiltration or infarction areas. The inferior vena cava showed partial thrombosis in the infra renal area. Her CT scan also showed a dilation of the superficial abdominal veins, and a partial opacification of the common iliac veins, the external and the internal iliac veins. The right common iliac vein was narrowed with a central filling defect, and the left common iliac vein was dilated with a thrombus attached to the wall. These are shown in the contrast-enhanced CT scans in Figure nine. The contrast-enhanced CT scans are shown in Figures 5, 6, 7, 8, 9 and 10.

## Discussion

aPLs are known to be associated with the occurrence of venous and/or arterial thrombosis. However, bleeding is rare in cases of APS [14]. Our patient was diagnosed with APS, with a recent history of norethisterone use, who was admitted to our hospital with severe menorrhagia. During admission, she developed thrombocytopenia and was found to have extensive IVC and bilateral common iliac thrombosis, which made the course of treatment challenging because it is difficult to use anticoagulation in a patient with heavy bleeding. Only a few cases of APS have reported simultaneous bleeding and thrombosis [3-8], and only a few of these cases have reported thrombosis induced by norethisterone when used by patients with an underlying risk factor for thrombosis [9].

APS is an autoimmune multisystem disorder of recurrent thrombosis and/or pregnancy losses that is associated with the presence of aPLs [2]. Primary antiphospholipid syndrome (PAPS) is the most common cause of acquired thrombophilia and accounts for 15 to 20% of all episodes of DVT [2]. All sites of the vascular bed may be subjected to thrombosis, large, medium and small, but the most common site is the deep venous system of the lower limbs and pulmonary embolism. Other reported sites of venous thrombosis include the pelvic, renal, mesenteric, portal, hepatic, axillary, sagittal veins and IVC [2]. There are several factors that may trigger the risk of thrombosis in APS including infection, trauma, surgery, withdrawal of oral anticoagulation [15] and drugs such as oral contraceptive pills. In this case study, thrombosis was possibly triggered by drug use (norethisterone). In women with previous or current thrombosis or an underlying risk of thromboembolism, when considering the use of norethisterone, the benefits of treatment need to be carefully weighed against the risks. If VTE develops after initiating therapy, the drug should be discontinued [13].

In patients with venous occlusive disease and recurrent fetal loss, testing for protein C, protein S and antithrombin III deficiency or the factor V Leiden and prothrombin mutation should be conducted according to the economics, test availability and the clinical likelihood. It is also useful to test patients with arterial occlusive disease for hyperhomocysteinemia [14]. In this case, our patient fulfilled the criteria for APS, making conducting these tests nonessential. Protein C and protein S tests were not conducted because she was on warfarin. Her tests for antithrombin III deficiency showed normal results, with her mild positive resistance to activated protein C (APC-resistance ratio) possibly due to anticoagulation. As for the factor V Leiden and prothrombin mutation tests, they were not conducted because they were unavailable at the hospital. Although there are no guidelines regarding monitoring or measuring the anti-Xa levels when low molecular weight heparin (LMWH) is used in patients with high thrombotic risk, like our patient, it must be considered to maintain therapeutic levels when available.

The treatment of acute venous thrombosis associated with APS is similar to the treatment of other forms of thrombosis. The treatment consists of anticoagulation with heparin, either unfractionated or low molecular weight, followed by warfarin, but then warfarin is used for secondary thrombosis prophylaxis indefinitely with the recommended INR of 2.5, while for recurrent thrombosis the recommended INR is 3 to 4. Other medications have been also used in well-anticoagulated patients who continue to have thrombosis, aspirin, hydroxychloroquine, a statin drug, IVIG and plasmapheresis [14]. Hydroxychloroquine has antithrombotic effects by inhibiting platelet aggregation and arachidonic acid release from simulated platelets [2]. Corticosteroids have no role in the treatment of APS. However, high doses of corticosteroids are usually given to patients with severe thrombocytopenia, hemolytic anemia and catastrophic APS [14].

Bleeding is a rare manifestation of lupus anticoagulant-antiphospholipid unless associated with coagulation factor deficiency, severe thrombocytopenia less than 30x10^3^/μL, anticoagulation overdose or the presence of an acquired prothrombin deficiency. Thrombocytopenia is a well-recognized feature of APS. aPL-associated thrombocytopenia is usually moderate without bleeding, and platelet levels fluctuate usually in the range of 100,000 to 150,000/μL, but are seldom low enough to be associated with bleeding [2]. The pathogenesis of thrombocytopenia in APS is unclear. Hypotheses include the binding of aPLs to platelet membrane phospholipid, B2-GPI/phospholipid complexes or coexisting antibodies to platelet membrane glycoproteins [2]. It was found that patients with APS associated with SLE more frequently exhibited thrombocytopenia than patients with primary APS (43 versus 21%) [2]. Thrombocytopenia does not preclude the occurrence of thrombotic complication of APS [1]. The treatment of thrombocytopenia is the same for idiopathic thrombocytopenic purpura [15], which is corticosteroids and other medications used for steroid-resistant thrombocytopenia, such as IVIG and danazol. Also, splenectomy or rituximab have been used in patients with refractory thrombocytopenia.

One research has found a two to four-fold increased risk of blood clots after plane travel due to low pressure, high altitudes and lower oxygen levels. This risk increases with the length of the flight, and patients with an underlying risk of thrombosis may be more vulnerable to disturbances in the clotting system during the flight. For our patient, the flight did not significantly contribute to her thrombosis because the flight was only two hours long and her INR was closely monitored [16].

## Conclusions

This case report is of interest to rheumatologists, hematologists and radiologists because we have found that the presence of bleeding and thrombocytopenia do not preclude the concomitant occurrence of thrombotic complications of APS. Norethisterone is normally safe to take, but it is not suitable for patients with an increased risk of DVT. Also, the simultaneous management of thrombosis and heavy vaginal bleeding is a challenge for clinicians since there are no evidence-based guidelines regarding the management of these patients.

## Consent

Written informed consent was obtained from the patient for publication of this case report and accompanying images. A copy of the written consent is available for review by the Editor-in-Chief of this journal.

## Abbreviations

APS: Antiphospholipid syndrome
IVC: Inferior vena cava
aPL: Antiphospholipid
VTE: Venous thromboembolism
DVTs: Deep vein thrombosis
PR: Pulse rate
BP: Blood pressure
RR: Respiratory rate
WBC: White Blood Cells
Hb: Hemoglobin
MCV: Mean Corpuscular Volume
MCH: Mean Corpuscular Hemoglobin
PT: Prothrombin Time
INR: International Normalized Ratio
PTT: Partial Thromboplastin Time
APC: Activated protein C
AST: Aspartate Aminotransferase Test
ALT: Alanine Aminotransferase Test
ALP: Alkaline Phosphatase
TP: Total Protein
ALB: Albumin
T.BILI: Total Bilirubin
D.BILI: Direct Bilirubin
LDH: Lactate Dehydrogenase
HCQ: Hydroxychloroquine
